# Modeling cardiac complexity: Advancements in myocardial models and analytical techniques for physiological investigation and therapeutic development *in vitro*

**DOI:** 10.1063/1.5055873

**Published:** 2019-02-05

**Authors:** Neal I. Callaghan, Sina Hadipour-Lakmehsari, Shin-Haw Lee, Anthony O. Gramolini, Craig A. Simmons

**Affiliations:** 1 Translational Biology and Engineering Program, Ted Rogers Centre for Heart Research, Toronto, Ontario M5G 1M1, Canada; 2 Institute of Biomaterials and Biomedical Engineering, Faculty of Applied Science and Engineering, University of Toronto, Toronto, Ontario M5S 3G9, Canada; 3 Department of Physiology, Faculty of Medicine, University of Toronto, Toronto, Ontario M5S 1A8, Canada; 4 Department of Mechanical and Industrial Engineering, Faculty of Applied Science and Engineering, Toronto, Ontario M5S 3G8, Canada

## Abstract

Cardiomyopathies, heart failure, and arrhythmias or conduction blockages impact millions of patients worldwide and are associated with marked increases in sudden cardiac death, decline in the quality of life, and the induction of secondary pathologies. These pathologies stem from dysfunction in the contractile or conductive properties of the cardiomyocyte, which as a result is a focus of fundamental investigation, drug discovery and therapeutic development, and tissue engineering. All of these foci require *in vitro* myocardial models and experimental techniques to probe the physiological functions of the cardiomyocyte. In this review, we provide a detailed exploration of different cell models, disease modeling strategies, and tissue constructs used from basic to translational research. Furthermore, we highlight recent advancements in imaging, electrophysiology, metabolic measurements, and mechanical and contractile characterization modalities that are advancing our understanding of cardiomyocyte physiology. With this review, we aim to both provide a biological framework for engineers contributing to the field and demonstrate the technical basis and limitations underlying physiological measurement modalities for biologists attempting to take advantage of these state-of-the-art techniques.

## I. INTRODUCTION

Compromised contractility of the heart is a major cause of death and decreased quality of life worldwide. Cardiomyopathies, including dilated, restrictive, or hypertrophic subtypes among others, are associated with reduced contractile or conductive function in the myocardium.^1^ These pathologies and others can often lead to heart failure (HF), affecting approximately 6.5 million patients over 20 years old in the USA alone, which is expected to rise to >8 million over 18 years old by 2030.^1^ From age 45 to 95, the overall lifetime risk of developing HF is between 20% and 45%, and the total yearly cost of HF was estimated to be over $30 billion (USD) in 2012.^1^ Heart failure can be caused by (epi)genetic inheritance, age, lifestyle, pharmaceuticals, or idiopathic factors and is difficult to treat effectively, as its causes are not always evident. Moreover, cardiomyopathy patients are at higher risk for a host of secondary pathologies or acute adverse events due to poor circulation. Various fibrillations such as atrial fibrillation (affecting over 30 million patients worldwide by itself), long- and short-QT syndromes, ventricular tachycardia, and other channelopathies stem from impaired pacing or electrophysiological conduction within the heart and contribute disproportionally to sudden cardiac death.^2–4^ To reduce the burden of myocardial pathologies, further study of the myocardium's functional unit, the cardiomyocyte (CM), is necessary.

## II. THE MYOCARDIUM IN CONTEXT

As the cell responsible for the beating of the heart, the cardiomyocyte (CM) is one of the most structurally and functionally specialized cells in the body. The relative proportion of cells in the heart remains a controversial issue, but cardiomyocytes make up 18%–33% of the human heart by cell number but 70%–80% by volume.^5,6^ The remainder of the human myocardium is composed mainly of mesenchymal cells such as fibroblasts (12%–58% by number) and endothelial cells (24%–54%), with small populations of resident macrophages and various progenitor cells; it also remains contentious whether relative cell populations vary by species.^5,6^ CMs are defined by the area in which they reside, which determines their precise function and electrophysiological profile. Nodal CMs are limited to the sinoatrial (SA) and atrioventricular (AV) nodes; atrial and ventricular cells also maintain phenotypic differences.^7,8^ The SA node consolidates inhibitory and excitatory nervous and hormonal input^9^ and generates an autonomous impulse to contract,^10^ which travels initially through the atria to reach the AV node. The AV node provides an electrical bottleneck between the atria and ventricles, affording a cohesive ventricular contraction as the contractile impulse diffuses through the ventricular myocardium and specialized Purkinje fibres in the septum.

**FIG. 1.**
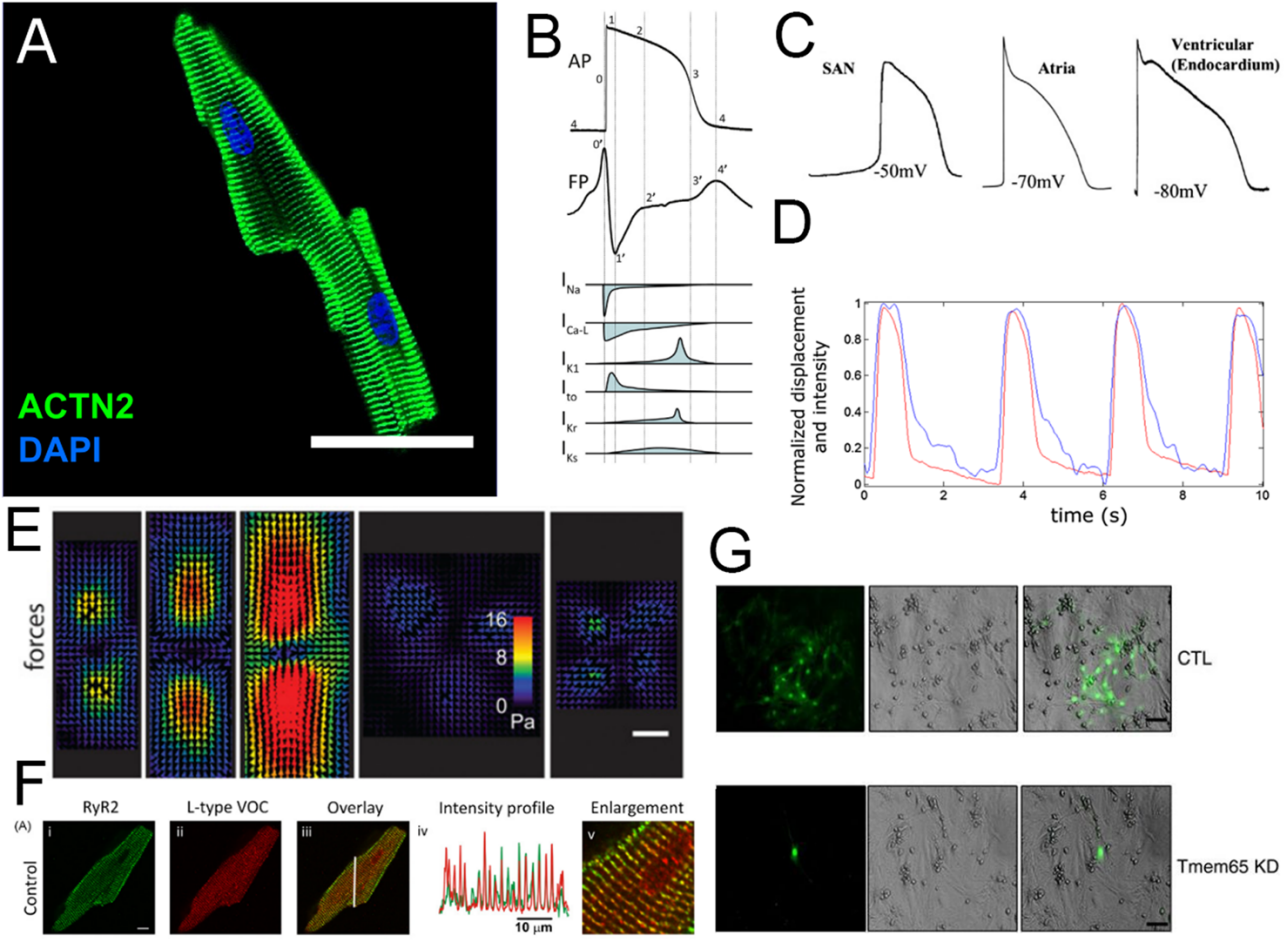
(a) An adult murine cardiomyocyte (CM), stained for sarcomeric α-actinin. Sarcomeres are continuous across the bundle of myofibrils that form the cell.^21^ Reprinted with permission from Ackers-Johnson *et al.*, Circ. Res. **119**(8), 909–920 (2016). Copyright 2016 Wolters Kluwer Health, Inc. (b) The cardiac action potential (top) is composed of four distinct phases, each with a specific ionic flux component (bottom). The cardiomyocyte produces a field potential (middle) that is highly correlated with the shape of its action potential.^22^ Reprinted with permission from Tertoolen *et al.*, Biochem. Biophys. Res. Commun. **497**(4), 1135–1141. Copyright 2018 Author(s), licensed under a Creative Commons Attribution 4.0 International License. (c) The resting membrane potential, and the precise shape of the action potential is specific to the CM subtype; nodal, atrial, and ventricular CMs have unique electrophysiological fingerprints.^8^ Reprinted with permission from Liu *et al.*, Adv. Drug Delivery Rev. **96**, 253–273. Copyright 2016 Elsevier. (d) The flux of contraction-enabling Ca^2+^ into the cytosol (black) is correlated with cell contraction strain (blue).^23^ Reprinted with permission from Ahola *et al.*, Ann. Biomed. Eng. **46**(1), 148–158. Copyright 2018 Author(s), licensed under a Creative Commons Attribution 4.0 International License. (e) Contractile CMs of different aspect ratios (3:1, 5:1, 7:1, unpatterned, and 1:1, respectively) deform compliant substrates allowing for the reconstruction of traction force vectors.^24^ Reprinted with permission from Ribeiro *et al.*, Proc. Natl. Acad. Sci. **112**(41), 12705–12710. Copyright 2015 National Academy of Sciences. (f) Transverse tubules responsible for initial Ca^2+^ inward flux (indicated by L-type Ca^2+^ channels, red) co-localize with sarcoplasmic reticulum (indicated by ryanodine receptor, green), which provides most of the cytosolic Ca^2+^ flux during a contraction.^25^ Reprinted with permission from Smyrnias *et al.*, Cell Calcium **47**(3), 210–223 (2010). Copyright 2010 Elsevier. (g) Gap junctions establish a functional syncytium, allowing HTTS (hydroxypyrene-1,3,6-trisulfonic acid, trisodium salt) dye propagation between cells. Knockdown of intercalated disc trafficking protein Tmem65 ablates gap junction formation.^26^ Reprinted by permission from Sharma *et al.*, Nat. Commun. **6**, 8391 (2015). Copyright Springer Nature.

The structure and function of the CM have been covered in depth elsewhere.^11^ Figure 1 provides a basic description of the CM morphology and functional readouts. Briefly, each CM is a bundle of myofibrils arranged in forms ranging from cylindrical to brick-like; myofibrils provide contractile power through sarcomeres, regularly interspersed ladder-like arrangements of the actomyosin complexes and associated proteins [Fig. 1(a)]. In general, thicker cells can be found in the ventricles and narrower, more cylindrical cells in the atria where less contractile power is generated. The CM contains very particular ion channel arrangements at the cell membrane (sarcolemma) and in sarcolemmal invaginations called transverse tubules (t-tubules). A 4-phase action potential (AP) initiates excitation of the CM; individual component currents [Fig. 1(b)] differ between CM subtypes (i.e., ventricular, atrial, and nodal), resulting in a different action potential waveform [Fig. 1(c)]. The longitudinal propagation of the action potential along the sarcolemma induces ionic calcium influx to the cell through voltage-gated (L-type) ion channels, triggering a larger calcium release through the ryanodine receptor (RyR2) from the sarcoplasmic reticulum. The released calcium induces the motor function at actomyosin complexes located at each sarcomere, initiating a contraction [Figs. 1(d) and 1(e)]. In a mature CM, the majority of cytoplasmic Ca^2+^ underlying a contraction is released from sarcoplasmic stores by the ryanodine receptor, through “dyads,” or t-tubule-sarcoplasmic reticulum couplings [Fig. 1(f)]. Action potentials, along with Ca^2+^ and various small molecules, pass between CMs at their poles through intercalated discs (ICDs), which provide tissue-level electrical coupling and mechanical signaling to modulate the cell function [Fig. 1(g)]. CMs also possess a unique metabolic profile that consists mainly of aerobic (oxygen-requiring) processes; this is given in detail more thoroughly in the section on metabolic characterization below (Sec. VI). After birth, CMs are virtually arrested in the cell cycle and rarely proliferate;^12^ the majority of new CMs are thought to emerge from mitotic CMs.^13^ The functional maturity of CMs is often assessed through correlative measures such as the relative expression of mature structural, contractile, metabolic, and ion handling protein isoforms.^8^ Similarly, cell responses can be assessed by transcriptomic methods during drug discovery;^14^ however, these are best coupled with functional metrics to assess the final physiological state of the cell. Pathology can occur in any aspect of the physiology of the CM with far-reaching implications, as cell signaling pathways, the cytoskeleton, and cell homeostatic processes are highly interdependent.

The majority of modeling work does not account for sex differences in SR Ca^2+^ handling and ECC,^15^ connexin expression^16^ (and thus ostensibly cell-cell propagation of impulses), or CM hormone responsiveness.^17^ Along with other differences in myocardial physiology, these lead to important sex-specific functional differences in cardiac electrophysiology and contractility and therefore predispositions to certain pathologies and pharmaceutical responsiveness.^18–20^ In general, much more work is required to characterize female-specific myocardial functionality and disease.

To elucidate pathological mechanisms and treatment options, a variety of models are used in cardiac research. Animal models provide high-level descriptions of pathologies and effects of treatments, but they carry several experimental drawbacks, most notably in physiological dissimilarity (i.e., protein expression patterns manifesting in electrophysiological, contractile, and metabolic functional differences) from humans. Furthermore, animals are expensive, sometimes ethically difficult to justify using, and prone to individual variability in response. Furthermore, data derived from animal studies can be difficult to interpret due to confounding variables given the high degree of complexity in a whole organism's response to an experimental condition.

To better isolate certain physiological mechanisms, extensive fundamental and preclinical work is undertaken at the cell and tissue levels. Single-cell myocardial models have been used in physiology and drug development for over 40 years.^27^ Early models have given rise to an array of cells used to model normal and pathological functions of CMs, including primary isolated neonatal and adult CMs, embryonic stem cell-derived or induced pluripotent stem cell-derived CMs (ESC-CMs or iPSC-CMs), and human embryonic kidney 293 (HEK 293) and Chinese hamster ovary (CHO) engineered cells. These cell types are covered in detail in Sec. III.

### A. Developing guidelines in cardiotoxicity modeling

Traditionally, preclinical research relied on the assessment of a pharmaceutical's proarrhythmic potential based on its propensity to directly induce Torsade de Pointes (TdP).^28^ TdP is a repolarization pathology with 20% mortality, and it is mostly associated with a single cardiac ion current [carried by the hERG K_v_11.1 ion channel that conducts the rapid delayed rectifier potassium current (I_Kr_)]. For largely unknown reasons, hERG is frequently subject to off-target effects by pharmaceuticals, and so, the hERG current is routinely genetically engineered to be stably expressed in HEK or CHO cells to assess TdP potential. Should the hERG current be unaffected, the development of the pharmaceutical would typically progress into an animal model. There were several limitations to this approach from a physiological or clinical perspective based on the complex composition of a contractile impulse in the CM due to ubiquitous off-target effects by channel-modulating pharmaceuticals. For example, blockade of certain sodium currents can delay repolarization by prolonging the QT interval, similar to TdP but in a hERG-independent manner.^29^ In contrast, verapamil inhibits hERG current and also has inhibitory activity against calcium currents and so does not show arrhythmogenicity. The failure to account for the net effect of a pharmaceutical, both directly on ion channel activity and indirectly on processes that eventually led to an aberrant ion channel function,^30^ meant that the cardiotoxicity of different drugs was often only recognized until well into clinical trials or even widespread clinical use as most commonly identified by meta-analysis. This issue began to be addressed using profiles of multiple ion channel effects in drug screens.^31^ The US Food and Drug Administration (FDA) simultaneously proposed and began to implement the Comprehensive *in-Vitro* Proarrhythmic Assay (CiPA) initiative to screen new drugs for TdP potential. CiPA consists of three components as follows:^32^

(1) A standardized assay of the drug against the activity of seven cardiac ion channels stably expressed in HEK/CHO models.
(2) A comprehensive *in silico* model to compute the net effect of the drug's activity on a human AP.
(3) Functional electrophysiological testing of the drug on a standardized human PSC-CM (hPSC-CM), followed by an *in vivo* animal electrocardiogram (ECG) if deemed necessary.

The benefit of CiPA will stem from standardized, high-throughput toxicity assays that are replicable between labs. This proposal requires a fully competent hPSC-CM model that recapitulates the exact electrophysiological profile of an adult cell, as many genes (such as those coding for KCNQ1 and KCNH2) that determine sensitivity to QT prolongation will only reach full expression in adults. CiPA is an ambitious but vital step towards effective drug safety screening and when completed will demonstrate the power of *in vitro* models in the pharmaceutical development pipeline. In the creation of biomimetic models and functional assays, however, CiPA represents just the beginning of next-generation screening. First, CiPA is effective in detecting TdP potential from direct effects on single cardiac ion channels,^33^ which provides an important checkpoint for most acute and unforeseen arrhythmias. However, human APs are known to be affected through myriad indirect effects regulated by intracellular scaffolding or trafficking,^34,35^ protein expression, metabolic state, and signal transduction pathways,^18,36–38^ and many non-TdP proarrhythmic compounds may not be detected through the standardized assays outlined in CiPA. Furthermore, drugs such as chemotherapeutics can induce severe damage to the myocardium over time^39^ which cannot be detected in an acute assay. Finally, a single model will not account for (epi)genetic variations within the population, even if all proarrhythmic genotypes were to be incorporated in a panel (a physical impossibility). Therefore, complex or rare toxicological mechanisms may escape detection until a catastrophic event in the lifetime of a drug, namely, clinical trial failure or post-release patient mortality. However, the tools being developed to realize the goals of CiPA are proving effective at detecting many physiological perturbations; these findings will be discussed in detail throughout this review. Moreover, the steps taken to achieve CiPA will allow for the development of other specialized screening models for these concerns.

### B. Advancing cell and tissue models and analytical tools through convergence science

**FIG. 2.**
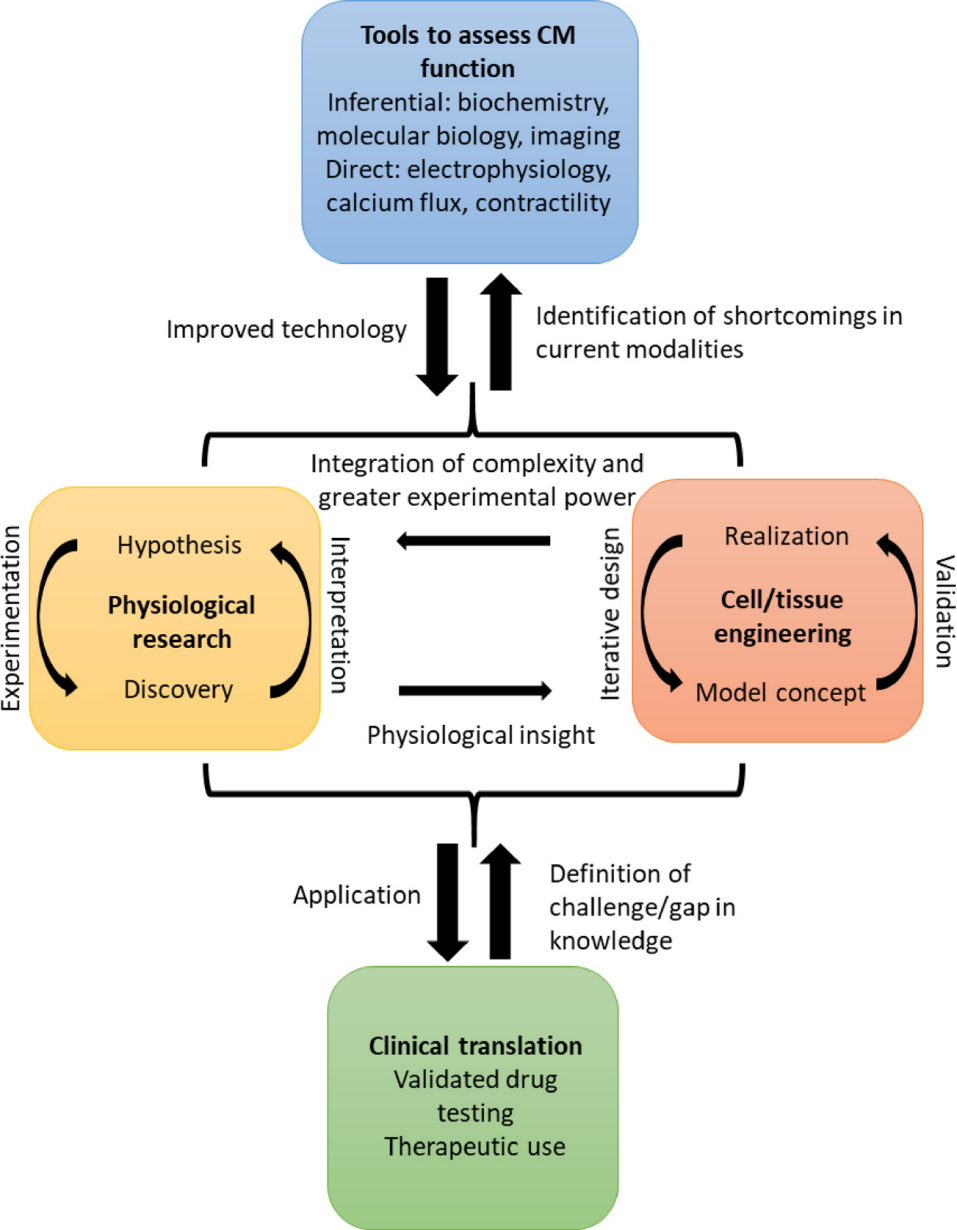
The role of advancing techniques to assess the cardiomyocyte function *in vitro* in the workflow to improved pharmaceutical testing and clinical outcomes. Both hypothesis- and discovery-driven research studies push each other forward, while informing iterations of cell and tissue models and devices, modalities, and techniques used in experimentation. The discoveries from fundamental science can then be translated to the clinic, while clinical findings can be used as a starting point for further fundamental inquiry.

While CiPA would represent a vast improvement in both FDA regulation and identification of torsadogenic compounds, both academic and industrial laboratories will ultimately benefit from a further mechanistic *in vitro* study to identify net cardiomodulatory effects exterior to TdP, which will ultimately save time and money, as well as potentially human and animal lives. Furthermore, these models and modalities developed for this purpose would be excellent for advanced physiological investigation, including novel targets and strategies for cardiovascular disease (CVD) therapies. There exist many long-standing physiological challenges in the cardiac health field such as how to predict cardiac responses to drugs, the mechanisms underlying ischaemia/reperfusion injury, how to stimulate myocardial regeneration, and how fibrillations arise and how they can be treated. These problems would benefit tremendously from increased engineering and analytical innovation, allowing for more detailed scientific questions to be asked and precisely answered. The relationship between technical development, investigation, and clinical translation in the cardiac space is mutually beneficial (Fig. 2). This review will discuss the current devices and techniques to model, capture, and analyze advanced metrics of the CM physiology and function. The applications, state of the art, and future directions in development and analysis will be discussed in detail.

## III. SINGLE CELL MODELS OF CARDIOMYOCYTE PHYSIOLOGY

**FIG. 3.**
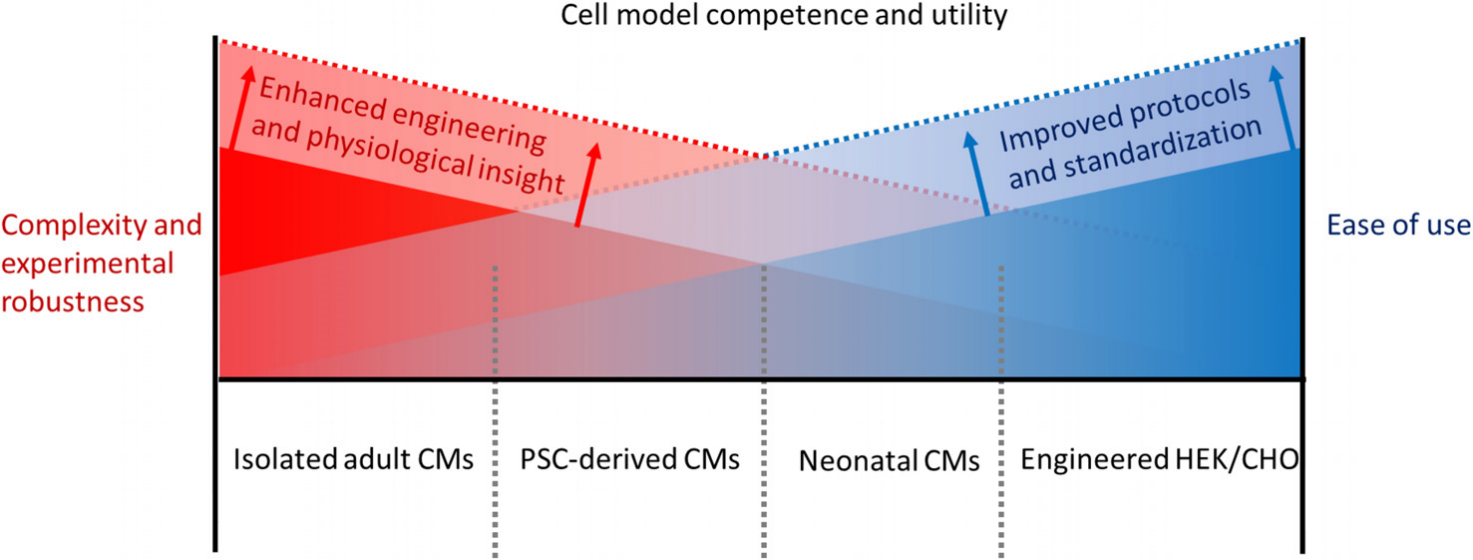
Negative correlation between the current usability of representative CM models and their final utility in advancing the field as a function of their ability to recapitulate complex and emergent CM physiological phenomena such as electrophysiology, contractility, signaling, and metabolism. By using improved functional characterization, which fuels improved experimentation, both the ease of use and scientific value and translatability can be increased.

*In vitro* cultures of single cells replicating one or more aspects of CMs *in vivo* represent the simplest unit of myocardium that can be responsively probed in an experiment. Single cells allow for the best isolation of an aspect of dynamic CM physiology short of non-physiologically responsive subcellular extracts. Specific functionalities including AP and Ca^2+^ flux, contractility, and metabolism all have CM-specific characteristics and complex determinants and respond in real time to internal signaling and external stimuli. The single-cell level of experimental control allows for a higher precision in elucidating mechanisms underlying a CM's response but sacrifices the complexity and translatability of a more complex system. Notable CM models, arranged by physiological completeness, include engineered HEK and CHO cells, primary isolated neonatal CMs, PSC-derived CMs, and primary isolated adult CMs. These cells occupy a continuum of physiological and experimental complexity (Fig. 3), where ease of use is balanced against translatability. The advantages and drawbacks of each model are discussed below. In general, there is no single ideal model: no cell type is able to recapitulate the full functional CM phenotype (comprising ion transport, signaling, metabolism, and contractility) in a sustained manner *in vitro.* This can be attributed to a lack of maturity in non-primary or non-mature CM models and current practical impossibilities in the culture of mature CMs.

### A. Genetically engineered HEK and CHO cells

Immortalized HEK and CHO cells are commonly used in many physiological systems. They are highly proliferative, are easy to culture, and can be transfected with high efficiency to express genes of interest. In the context of CM modeling, HEK and CHO cells are most commonly used to express one specific ion channel, such as hERG, at a time.^40,41^

There are limitations to the use of HEK and CHO cells. Their use in CM models does not recapitulate most of the defining aspects of CM physiology, including morphology, full action potentials, voltage-induced calcium flux, contractility, and metabolic organization, and substrate usage profile. For this reason, HEK and CHO cells are not routinely used in CM physiological studies (i.e., attempting to probe CM-specific responses to stimuli or the roles of biomolecules in the function of CMs) beyond single-channel quantitative drug toxicity studies.

### B. Primary neonatal cardiomyocytes

Primary neonatal CMs isolated from mice and rats are ubiquitous in physiology and tissue engineering studies. Used primarily for their relative robustness in culture and cost, they are highly characterized and thus offer a simplified, but useful, platform to study and innovate. Neonatal CMs are relatively easy to isolate, and there are many robust protocols for doing so.^42,43^ Most involve the isolation of cardiac tissue from 1 to 3-day old pups, mincing to increase the surface area for subsequent digestion by trypsin and collagenase. There is no requirement for perfusion of the cardiac tissue, and high purity of cardiomyocytes can be achieved by a pre-plating step to remove fibroblasts. Neonatal CMs adhere with relative ease to 4% gelatin, and they can be plated on a variety of cell culture dishes. Neonatal CMs have great potential for genetic manipulation, generally with lentiviral-mediated transduction, and can last up to several weeks in culture. Distinct sarcomeres can be achieved after approximately 1 week in culture, showing signs of physical maturation.

As these CMs are transiently proliferative in culture (ostensibly via expression of the Tbx20 transcription factor,^44^ which seems to be a main regulator of the CM cell cycle^45,46^) before eventual *in vitro* maturation, they can populate a 2D culture or microtissue. These cells are robust and hypoxia-insensitive when compared to more mature analogs due to a higher relative glycolytic capacity. However, as previously implied, their robustness in culture is counterbalanced by their immature morphology, contractility, maturity-linked protein isoform expression profile, and electrophysiology.

### C. Pluripotent stem cell-derived cardiomyocytes

Directed differentiation of pluripotent stem cells, both induced pluripotent and embryonic, can result in populations of >90% pure cardiomyocytes (PSC-CMs). Since the initial derivation of PSC-CMs, our understanding of the underlying mechanisms in CM differentiation has rapidly advanced. PSC-CMs offer the possibility of large yields of standardized cells for therapy and physiological studies. Moreover, as they can be derived from patients, PSC-CMs can be used to model physiological responses to a panel of drugs, on a disease- or even patient-specific basis. Validated commercial iPSC-CM sources are available from companies including Pluriomics, Stem Cell Technologies, and Cellular Dynamics, while validated commercial differentiation kits (e.g., those from Gibco and Stem Cell Technologies) are based with a slight variation on common protocols in the literature which provide replicable high yields.^47–51^ Current experimentation is revealing differentiation protocols specific to regions of the heart (i.e., ventricular, atrial, and both nodes).^52,53,54^ In general, nondirected commercial protocols generate mainly ventricular CMs, similar to neonatal CM isolations.

Within recent years, the functional maturation of PSC-CMs has been greatly improved, according to morphological, electrophysiological, and protein isoform expression metrics.^29,55–59^ These maturation protocols are often replicable between labs and PSC lines; however, the most advanced single-cell models still only generate a fraction of the contractile force of an adult CM,^57^ while hPSC-CM microtissues are approaching native adult myocardial cross-sectional force production.^60,61^ Furthermore, even after differentiation, optimal culture conditions still have not been established for PSC-CMs.^62^ Regardless, based on measurements of contractility and multi-electrode array (MEA) field potential, PSC-CMs are predictive of drug toxicity,^63,64^ and unlike primary CMs, they can be reasonably assessed in parallel and high-throughput assays. However, the “black box” underlying functional maturation remains; the mechanistic insight into cardiotoxicity from these models will not be widely accepted until the physiological maturation pathways of CMs can be further elucidated.

### D. Primary adult CMs

Primary adult CMs, usually from mice, rats, or rabbits, can be isolated for physiological studies through Langendorff or non-Langendorff perfusion protocols. In Langendorff perfusion isolation, a heart is reverse-cannulated through the aorta, and an anticoagulant solution (usually heparin or EDTA) is passed through the heart to clear blood, before flooding the heart with a collagenase-containing digestion solution. The pressure of the retrograde perfusion allows for penetration of digestive enzymes deep into the myocardium. This method generally offers a higher yield of viable cells than alternative protocols but is technically difficult to perform by untrained personnel. Langendorff-free isolation protocols are usually performed on mechanically separated pieces of myocardium bathed in digestion solution, although a recent protocol has shown considerable success using needles to deliver digestion solution to a clamped heart, in order to maintain pressure and perfusion efficiency.^21^

The advantage of primary adult CMs in physiology and toxicology is in high similarity of these cells to adult human CMs. These cells maintain most physiologically relevant characteristics in the morphology, metabolism, and protein expression patterns. However, there are several drawbacks to the use of primary adult CMs. These cells are difficult to culture, with high initial and sustained mortality in culture and rapid de-differentiation *in vitro* (i.e., recapitulating the morphology and function of a much younger CM or even a fibroblastic cell). For this reason, there has been no successful organized tissue culture (microtissue or higher) of primary CMs. Finally, even in high-quality cultures, there are species-specific differences in the morphology (cell size, binucleated population, etc.), contractility, and electrophysiology. Therefore, exact physiological findings from a different species will never be directly translatable to clinical practice. Despite these limitations, due to their recapitulation of adult CM behaviour, these cells are invaluable in physiological study.

## IV. DISEASE MODELING IN CELLS

Along with the prediction of pharmaceutical cardiotoxicity, CM models offer significant promise for elucidating mechanisms of disease and developing novel treatments. In general, cardiomyopathy manifests in cellular hypertrophy and dysregulation of other constituent cell [i.e., fibroblasts (FBs), endothelial cells (ECs), and immune cells] functions based on the mechanism of the pathology in question. This disrupted homeostasis results in altered responses to nervous and hormonal cardiac regulation in disease-specific patterns; treatment goals are therefore to regain normal electrophysiological and contractile function and response to external stimuli without overworking the heart. As these conditions progress into pathology over months or years, the complexity of the condition is difficult to model *in vitro* or even in *in vivo* animal models, leading to difficulty in translating model studies to the clinic. As such, the simplest method of modeling pathology is through the use of primary or PSC-derived cells originating from individual animals or patients with a genetic disease (such as Barth syndrome^65^) or induced disease models (such as diabetes^66^). Thorough reviews of cardiomyopathic phenotypes *in vivo* and their replication *in vitro* can be found elsewhere.^67,68^

A basic and robust method of adding or removing genes of interest can be found in the Cre-LoxP system, whereupon a plasmid can be inserted at the ROSA26 locus, tamoxifen inducible promoter, or *MYH7* or *cTNT* for germline, inducible, or myocardium-specific expression or knockout, respectively.^69^ These methods are generally carried out in animal models from which primary cells can be used for *in vitro* experiments to complement *in vivo* physiological measurements.

Alternatively, the use of various CRISPR/Cas9-compatible transfection techniques both *in vivo* and *in vitro* represents a rapidly advancing area of research. In whole animals, as with pre-CRISPR-era transduction techniques, lentiviral or adeno-associated viral transduction methods are standard due to their high efficiency (ca. 80%); these viruses are typically injected intraperitoneally or in the tail vein of mice. These methods however commonly cause off-target effects and are highly immunogenic.^70,71^ The *MYH7*-Cas9 promoter can be used to reduce non-myocardial transfection with AAV9. *In vitro*, primary cardiomyocytes can be most efficiently transduced via viral vectors; the use of electroporation, microsomal vectors, or chemical poration using lipofectamine 2000 for CRISPR/Cas9-mediated modifications is viable if less efficient methods (the latter is the most reliable at ca. 30% efficiency).^70^

There are excellent reviews on the use of CRISPR/Cas9 for genetic manipulation in cells and animals.^72^ The suite of techniques is highly suitable for gene knockdown,^73^ editing,^74^ or insertion. For near-ubiquitous expression in PSC-derived cultures, clonal selection using green fluorescent protein (GFP), *tdTomato* or other target genes allows for a higher percent efficiency at the final post-differentiation CM stage, ideal for a genetically homogenous tissue. Transgenic animals have recently been bred with constitutively expressed Cas9, allowing for simple manipulation of cardiac-specific genes.^75^ Finally, short hairpin and small interfering RNAs (shRNA and siRNA, respectively) can be used to temporarily knock down the expression of a specific gene. siRNA is simpler to produce and produces more transient and off-target effects, while shRNA is more stable and specific to its target. Both RNA interference methods can be delivered to the cell by various methods as previously discussed, including lentivirus, AAV, microsomal, or direct entry methods.^76^

In all of the genetic engineering techniques discussed here, development is continually focused on increasing specificity and efficiency, while allowing for a definable effect size and a lack of immunogenicity. In general, CM culture requires more mature models that are culture-stable, replicable between batches, and able to replicate the complexity of the native CM.

## V. ADDING COMPLEXITY: REPLICATING MYOCARDIAL PHYSIOLOGY WITH TISSUE CULTURE MODELS

### A. Trends in advancing cell and microtissue culture

In recent years, research has firmly established the advantages of microtissue-scale isolated neonatal or PSC-CM-containing models in recapitulating the function and complexity of the myocardium due to the positive contributions of dimensionality, cell-cell interactions, external biochemical cues, and mechanical and electrical stimulation. There are many excellent reviews on the current state of the cardiac tissue engineering field;^77–79^ here, we will briefly cover trends and potential future directions that can be further developed upon. The basic necessities of monolayer culture will be discussed, followed by 3D culture paradigms, co-culture attempts, and stimulation using exogenous chemical, electrical, and mechanical cues. Finally, novel biomaterials to enhance CM culture will be briefly discussed.

The simplest reduction of *in vitro* modeling efforts posits that by replicating the myocardial niche, complex culture systems allow for a facsimile of the *in vivo* maturation process. One of the first steps in replicating the myocardium is to enable or promote cell-cell interactions. CMs are highly reliant on other CMs for AP propagation and mechanical signaling; their intercalated discs (ICDs) include gap junctions that allow for the passage of voltage and calcium waves and small molecules. ICDs also contain adherens junctions and desmosomes that allow for healthy signaling within and between CMs.^80,81^ The functional syncytium is essential to the normal CM function, as the cell transduces Ca^2+^ flux through PI3K, PKA, PKC, Wnt, and NFAT signaling pathways, among others.^82^ In single cells without any sort of regularized AP/Ca wave, physiology will rapidly become aberrant. Even a single cell-cell connection between an adult CM and one of the juvenile phenotypes allows for significant functional maturation of the latter.^83^ Early attempts at myocardial tissue engineering were often undertaken using neonatal CMs; most new efforts make use of PSC-CMs.

Cultures of PSC-CM microtissues are typically assessed relative to *in vivo* measurements for organization, morphology, biochemical markers, and contractility, although they can also be compared to their isolated *ex vivo* counterparts.^84^ Differences between a given microtissue's function *in vivo* and *in vitro* are unclear due to the inability to culture adult isolated CMs or myocardium effectively as benchmarks. The development of improved *in vitro* benchmarks for single PSC-CMs and their tissues will be essential for continued advancement of the field, in order to make PSC-CM models standard for physiological, pharmaceutical, and therapeutic adoption.

### B. Co-culture

The CMs in the myocardium are balanced by a complement of fibroblasts, vascular smooth muscle cells, endothelial cells, and a small but vital contingent of resident immune cells. These cells are vital for maintaining functional myocardial physiology, including mechanical support and perfusion sufficient to allow for concerted beating. As our understanding of myocardial physiology advances, it is clear not only that a functional model of the myocardium will require a diversity of cell types but also that the superstructure of the construct closely resembles the native arrangement. Significant efforts are currently focused on both understanding and implementing the role of co-culture in the development, functioning, and pathology of the myocardium.

Ostensibly, the most important consideration in constructing a myocardial co-culture platform is in its cell type composition and its relative proportions in the model, which varies significantly by species and by age. For example, an adult mouse heart contains ca. 56% myocytes and 26% fibroblasts by number, but an adult rat heart contains ca. 26% CMs and 63% fibroblasts by number. Fibroblasts, endothelial cells, and vascular smooth muscle cells enhance CM maturation and tissue-level function including contractility and conduction. The fibroblast is best known for maintaining the ECM content of the heart, often to pathological levels in instances of cardiac fibrosis. The ECM produced by fibroblasts seems highly dependent on the cellular makeup of the culture,^85^ by mechanisms not yet fully elucidated. However, fibroblasts are gradually being identified as workhorse cells with diverse and vital functions. Fibroblasts can form gap junctions with cardiomyocytes and have been shown to enhance electrical conduction and physiological remodeling in the heart,^86^ as well as increased contractile force production;^87^ they also impact conduction based on their relative population within tissue constructs.^88^ Furthermore, they exert significant paracrine and autocrine effects.^89^ Inclusion of endothelial cells in co-culture has been shown to enhance the CM electrophysiological function and transcriptional patterns.^90^ Similarly, CM co-cultures with fibroblasts and microvascular endothelial cells were better at predicting inotropic drug effects.^91^ Mesenchymal stem cells in the myocardium *in vivo* may also enhance the tissue function.^92^

Although development of models containing fibroblasts, vascular cells, and endothelium is well underway, few models include immune cells. Immune cells form a relatively small proportion of the myocardium and often can be difficult to culture such that desired cell phenotypes are exhibited. However, a growing body of evidence suggests that they are important to both healthy and pathological myocardial functions, not in small part due to their paracrine function. CD3^+^ T-lymphocytes and CD68-KP1^+^ macrophages have been found to be more common in atrial fibrillation (AF) patients,^93^ and immune infiltration is also associated with AF and fibrosis, although causative mechanisms have not yet been definitively established.^94^ There also exists a resident population of cardiac macrophages which persists through life which is important in cell stress and damage responses, as well as remodeling.^95^ Together, these findings suggest that the myocardial cell balance is specific to phenotype and essential in properly recapitulating homeostasis.

### C. External biochemical techniques to enhance model patency

By taking advantage of well-known biochemical insights, researchers have dramatically enhanced CM survival and physiological relevance to *in vivo* systems. Many of these measures are excellently summarized elsewhere.^96^ The use of M199-based media is near-ubiquitous for primary CM cultures due to its near-physiological levels of many ions and glucose. Many PSC-CM media use an RPMI 1640 base with added supplements; however, the subphysiological concentrations of the divalent cations important in membrane integrity and signaling homeostasis and the superphysiological concentration of phosphate important in metabolic homeostasis may prevent full realization of the mature CM phenotype. However, the strategic use of biochemical supplements enhances CM culture. The use of additional creatine-carnitine-taurine (CCT) maintains the metabolic function and thus homeostasis,^96^ while the use of insulin-transferrin-selenium (ITS),^21,97^ especially with a lipid suspension supplement, can replace media serum supplementation and therefore avoid serum toxicity and batch-to-batch variation.

Although co-culture (see above) represents an exciting means of harnessing paracrine and direct cell-cell contact to regulate the CM function, non-CM cells from isolation or nonspecific differentiation can easily outgrow CMs and obfuscate physiological metrics of interest or otherwise add confounding variables to a study. Certain protocols will utilize media supplementation of the chemotherapeutic cytarabine (ara-C) to inhibit DNA replication in proliferating cells, which minimally affects quiescent CMs.^98^ Alternatively, many PSC-CM cultures use media glucose depletion to enhance metabolic maturity and cell purity, as CMs can survive on lactate and lipid substrates^24,99–101^ (discussed further in Sec. VI). It is likely that paracrine signals could be used to enhance existing culture protocols;^102^ the use of conditioned media may provide initial mechanistic data in this regard.

### D. 3D culture devices

For physiologically relevant applications, the most promising CM cultures have been developed in complex 3D systems, which can be integrated with co-culture and stimulation. Relatively simple 3D cultures are amorphous,^90^ relying on cellular reorganization of existing matrix proteins or *de novo* secretion of ECM from chemically linked cells.^103^ More anisotropic and structured bioreactors have also been developed, usually mimicking the contractile bundles found in the myocardium. Standout examples include iterations of Biowire,^104,105^ including a version that includes perfusion mimicking CM-capillary organization.^106^ Other wire-based systems^110–112^ or bundles^60^ show an advanced myocardial function; the latter example by Jackman *et al.* recapitulates a substantial portion of both the electrical conduction speed and peak force production of adult myocardium. It is likely that due to the high degree of myocardial physiological specification allowed by these systems will cause them to be continual candidates for improvement in the future.

### E. Electrical and optogenetic stimulation

The regular electrical impulses to contract that propagate through myocardium *in vivo* are important regulators of calcium homeostasis, which influences virtually all aspects of cellular physiology. Single cells, whether adult or neonatal/PSC-derived, can be made to beat spontaneously under the proper culture conditions. In heterogeneous colonies of neonatal or PSC-CM cells, populations of nodal cells can initiate an AP that paces the colony; in a large enough population, a propagating AP can circulate continuously throughout a colony; this is the basis for current fibrillation-in-a-dish models.^53^ These impulses can be largely overridden and a more regular and reliable beating pattern obtained by using electric field stimulation in a dish equipped with two electrodes (usually platinum wires, carbon rods, or printed gold electrodes) that pass current through the cell culture media. Laboratory pulse generators are often used to provide basic square-wave impulses; existing reviews and protocols for engineered microtissues provide a range of parameters.^84,110–112^ Both monophasic and biphasic pulses are used in different studies due to introducing experimental complexity. Monophasic pulses are prone to inducing electrolysis and thus producing reactive oxygen species (ROS) and introducing pH gradients in culture, while biphasic pulses hyperpolarize cells and can interfere with AP propagation.^113,114^ reactive oxygen species (ROS) production in experimentation can be the product of cell metabolism imposed by increased contractility^115^ or media electrolysis; this may confound experimental analysis due to direct toxicity and the impact of ROS on a variety of signaling pathways.

Electrical stimulation is traditionally limited by electrolysis at the positive and negative poles. Various attempts to minimize electrolytic end-product toxicity have included inert electrode compositions such as platinum, carbon, and gold, as well as salt-bridges leading from isolated primary electrodes to secondary ones in the culture.^112^ Alternatively, genetic engineering efforts have shown success at incorporating the optogenetic channelrhodopsin-2 nonspecific cation channel into CMs^119–121^ to trigger action potentials upon blue light stimulation. This modality has been incorporated into several preclinical screening platforms (discussed below) and may prove to be of broad interest in both physiological and therapeutic development applications. Large and complex microtissues or small constructs in complex culture systems that are too thick to penetrate with one-photon could potentially benefit from multiphoton optogenetic stimulation and concurrent monitoring techniques developed for cortical purposes.^119^ Furthermore, optogenetics allow for spatial resolution in a single culture, allowing for region-specific waveform modulation, specialized repolarization gradients, and other biomimetic features.^114^ In general, it is likely that due to the inertness of optogenetic relative to electrical field stimulation, as well as the modularity of the technique, optogenetics will become the default choice for CM stimulation.

### F. Mechanical cues

CMs are exposed to a unique mechanical niche in the myocardium. They experience structural anisotropy and age- and disease-specific stiffness due to the surrounding ECM. CMs are subject to continual mechanical strain and shear stress as the myocardium contracts, as well as passive tension to the contraction by surrounding cells. Mechanosensing occurs through intercalated disc^80,81^ and focal adhesion associated proteins,^120,121^ titin-associated proteins,^122^ and stretch-activated ion channels^123^ and profoundly affects the physiology of CMs.

One of the most-studied mechanical effects on CM maturation and function is structural anisotropy. In studies using electrospinning, printing, and/or lithographical approaches that mimic nanotopographic aspects of the myocardial ECM, or otherwise constrain CMs to a mature (ca. 7:1–9:1) aspect ratio, CMs have been shown to respond strongly to anisotropy reminiscent of the myocardium.^124–128^ Furthermore, continual mechanical sensing by the CM means that the use of dynamic nanotopographies may be of use in modeling myocardial development or pathological progression.^129,130^ CMs are sensitive to dynamic and, to a lesser extent, static mechanical strain.^111,131,132^ Stiffness^121,133–135^ and passive loading^105,136^ are also important in in the short term ostensibly through mechanical and autocrine or paracrine signaling, while long-term changes in the cytoskeleton often represent a response to pathological stiffness or strain.^134,137–139^

### G. Novel biomaterials to enhance tissue culture

Biomaterial engineering is rapidly improving culture conditions for single CMs to complex microtissues. By mimicking myocardial chemistry, anisotropy, and elasticity, as well as integrating sensors, the development of cardiac muscle models has been considerably enhanced. For the purposes of tissue engineering, estimates of the stiffness of adult myocardium are 10–12 kPa at the start of diastole.^79,140,141^ The use of poly(dimethylsiloxane) (PDMS) and hydrogels is ubiquitous in myocardial TE applications due to their relative ease of fabrication, mechanical benefits to cultured cells, and chemical modularity. In fact, due to their contractility, neonatal and PSC-CM microtissues tend to partially detach from glass and polystyrene culture surfaces of stiffness in the gigapascal range, while better adhesion occurs with a more physiological stiffness. Hydrogels for CM microtissues are composed of a variety of materials, including poly(ethyleneglycol), hyaluronan, gelatin, gelatin-methacrylate, and alginate. Many of these efforts use conjugated, defined attachment moieties,^141,142^ the most common of which are synthetic peptides corresponding to the integrin attachment motifs of myocardial ECM proteins. The RGD sequence, usually the form of GRGDS or similar, is found in collagens, fibronectin, fibrins, and laminins^143,144^ and is the most common attachment peptide used in synthetic myocardial scaffolds.^124,125,145–147^ As the role of the full complement of the ECM in cell maturation and function is increasingly being recognized, due to signaling from different integrins and other cell adhesion receptors,^148^ attempts to use other attachment peptides, such as the YIGSR sequence specific to laminins, have shown favourable results.^143,146,147^ Finally, the incorporation of conductive materials such as graphene and carbon nanotubes to assist in electrical propagation and maturation has demonstrated measurable success.^81,149–151^

## VI. PHYSIOLOGICAL METRICS OF THE CELL AND TISSUE: METABOLISM, PROTEIN EXPRESSION, AND CELL STRUCTURE

Due to the enormous energetic demands of both continual counter-gradient ion cycling and force production, the myocardium consumes more adenosine triphosphate (ATP; the general-purpose energetic currency of the cell) per unit mass than any other tissue. The average adult heart will turn over 12–20 kg of ATP per day, and a CM will use more ATP in ∼10 s than can be contained within itself.^151^ Moreover, a human can increase cardiac output by 7-fold by increasing the cardiac metabolic rate by 10-fold.^152^ In the realm of metabolism, this represents a remarkably efficient scaling process. To satisfy these demands in performance, the myocardium relies almost solely on oxidative metabolism, which accounts for 90%–95% of ATP production. Nonoxidative metabolism in the heart is minimal, and it is mostly used to produce precursor substrates for further oxidation. The metabolic substrates of choice of the heart are 40%–90% lipids, 10%–15% ketones, 10%–40% glucose, 10%–30% lactate, and 1%–2% protein.^154–156^ There is a large degree of variability depending on transient hormonal and exercise states, with long-term differences by animal and life stage.^154,155^ Rodents and other small animals, along with very young and older humans, use significantly more glucose and less lipid than adults,^157^ and so, lipid usage is an excellent metric of health and maturity of human myocardial tissue. The biochemistry of these processes has been excellently reviewed by others previously.^154,158^ Although efficient in non-oxygen-limiting conditions, this metabolic scheme carries a serious drawback. The biochemical pathway underlying lipid catabolism is inherently oxygen-wasting compared to other substrates, and the reliance on oxygen in general means that any blockage, transient or chronic (such as embolism, thrombosis, and stenosis), can result in serious and permanent myocardial damage.

### A. Analyzing metabolic flux through respirometry and spectrometry

One of the simplest and most cohesive metrics of functional metabolism is the rate of oxygen consumption (MO_2_), produced by mitochondrial oxidative phosphorylation and beta-oxidation. As different substrates require different amounts of oxygen to fully oxidize, this rate can be coupled with the CO_2_ measurement to obtain a respiratory quotient or the metabolic signature of substrate preference. Basic cell respirometers have long been used with cardiomyocytes or their isolated mitochondria, assessing both MO_2_ and fluxes through different pathways with different substrates and small molecule inhibitors. Arguably, the most ubiquitous and advanced cell or tissue respirometry systems are found in the Oroboros O2k and Agilent Seahorse platforms. Both systems are extremely sensitive; Oroboros offers high customizability for complexed and novel measurements of paired samples, while the Seahorse platform is optimized for resolution and high-throughput measurements of several ubiquitous metrics of metabolism. The choice of platform therefore likely depends mainly on whether the experiment is fundamental or clinical/preclinical in nature. Standard protocols for measuring the most common respirometric indicators with appropriate controls can be found in a cohesive review published by Pesta and Gnaiger,^159^ and mitochondrial-specific protocols have been made available by Lanza and Nair.^160^

Given the effect of age and metabolism in regulating the function of a cell, ROS indicators provide valuable clues into incipient pathological phenotypes and aberrant signaling pathways. Fluorescent detection of mitochondrial superoxide generation through superoxide dismutase and horseradish peroxidase-coupled Amplex Red^161^ is a representative measurement of cellular ROS production and should be balanced with the measurement of available cellular glutathione pools,^162^ protein thiols,^163^ and their respective fluxes (such as in an experimental panel by Banh *et al.*^164^)

Mass-spectrometry (MS)-enabled proteomic,^165^ metabolomic,^166^ and lipidomic^167^ profiling is quickly becoming standard for *in vivo* physiological investigation and will likely soon be commonplace in characterizing CMs and myocardial constructs. For proteomic purposes, whole-protein analysis or trypsin processing can be used based on the resolution required.^165^ The proteomic effects of various pharmaceuticals^168–171^ or disease states^172^ can be compared; artificial constructs could also be compared to native tissue benchmarks to assess their maturity state. For insights into cell signaling pathways, typically of interest in a disease state or pharmaceutical response, phosphoproteomic profiles can be ascertained using titanium dioxide bead enhancement of phosphopeptide signals.^173^ Additionally, normalization between samples can be enhanced by using tandem mass tag (TMT) kits to label samples isotopically for pooling before analysis to prevent artifacts during sample preparation.^174^ Finally, enrichment of post-translational modification (PTM) signals can be used using antibodies to gain profiles of that PTM within the cell, such as global ubiquitination^175^ or glycosylation.^176^ Similarly, the use of matrix-assisted laser desorption/ionization (MALDI) or other MS imaging modalities allows for the imaging of spatially precise MS profiles^167,177,178^ that could greatly contribute to thorough characterization of microtissues, especially those in 3D.^179^

## VII. MICROSCOPY FOR FUNCTIONAL INSIGHTS

For qualitative or various semiquantitative metrics of the cell morphology and protein localization and expression, widefield or confocal fluorescence microscopy on fixed cells or tissues is a virtual requirement for most physiological and tissue engineering studies. The difference in disease- or maturity state-specific expression or organization of proteins or cellular processes allows for insights into underlying physiological mechanisms. Morphological cues including sarcomeric spacing and alignment, cell circularity and area, and nuclear alignment form a basis for the assessment of CM maturity,^106,125,129^ and advanced physiological insights can be gained by 3D reconstruction of a confocal Z-stack.^180^

There also exist many live fluorescent dyes for observing dynamic physiological processes in real time. In general, these protocols are straightforward, exert minimal phototoxicity, and can be used over extended time scales. However, dyes can be expensive and/or nonspecific, require a microscope incubator, and may disrupt the normal physiological function, thus confounding physiological interpretations. Commonly used live dyes for use in CMs include LifeAct for dynamic actin staining in real-time,^181^ Mitotracker for mitochondrial imaging,^182^ hydroxypyrene-1,3,6-trisulfonic acid, trisodium salt (HTTS) dye to identify functional syncytial gap junctions between cells,^26^ mtKeima dyes for mitochondrial autophagy,^183^ and myofibrillar dyes for CM structural maturation and remodeling.^184^ The advent of super-resolution microscopy has enabled spatial resolutions better than the diffraction limited resolution of optical lenses through a variety of different functioning principles; they are discussed thoroughly elsewhere.^185–188^ Most of these modalities are amenable to live dyes, with the caveat that long capture times limit their application to fast cellular events and that the considerable data burdens produced by these modalities can require considerable computational power for analysis. Only a few studies to date have taken advantage of super-resolution microscopy in the context of CM engineering or physiology due to these limitations.^189,190^

## VIII. ELECTROPHYSIOLOGICAL MEASUREMENTS

The action potential is a depolarizing ion gradient produced by the sequentially coordinated action of carefully regulated ion channels along the cell's membrane. In most CMs, an AP enters the cell from a gap junction at one extremity and leaves through the other, traveling through myocardium at a velocity of 0.3–1.0 m/s.^58^ In nodal or otherwise spontaneously contracting CMs, APs can be generated *de novo*; in fibrillation pathologies, APs can also be spontaneous or can arise from local recirculation of a previously conducted AP. As previously discussed, the action potential stimulates the release of calcium from L-type calcium channels found in t-tubules, which allows for massive calcium release from the SR. The functional AP represents a summation of many component currents, each the product of a specific ion channel; the AP can be measured in whole or as an aggregate probed through the separation of each component using specialized electrical techniques and specific ion channel inhibitors.

The first modality developed to quantitate APs, and still arguably the most versatile and precise, is the patch-clamp technique. In CMs, whole-cell patches and perforated patches are used to measure the electrical activity across the entire cell's membrane.^191^ Cells are current-clamped to measure the change in membrane potential during an AP and are voltage-clamped to measure the component currents of an AP. In both whole-cell and perforated patches, a glass recording pipette uses suction to form a gigaohm seal with the membrane; in the whole-cell patch, the enclosed membrane is suctioned away to form a continuous interface between the cell and electrode, while in the perforated patch, the local membrane is partially permeabilized to monovalent ions using poration agents, usually antibiotics. The perforated patch maintains the integrity of the cytoplasm and prevents Ca^2+^ and molecular leak to or from the pipette, preventing kinetic changes to Ca^2+^ or nucleotide-sensitive ion channels.^192,193^ Although the patch-clamp is the gold standard for sensitivity and versatility in assessing electrophysiological functions, it carries several limitations: it is low-throughput, especially when assessing multiple parameters; it is labour intensive, requiring careful equipment and cell preparation; and finally, patch-clamping is technically difficult and requires a skilled operator to generate reproducible data. Each cell must also be clamped similarly with respect to time patched, temperature, pH, and exposure to small molecules or hormonal inhibitors or agonists, to generate replicable data.

Attempts to reduce the skill curve and issues of throughput and replicability are being addressed by novel technologies. Planar patch-clamp systems^194^ and automated multi-well patch clampers^195^ allow for simultaneous, replicated recordings across multiple conditions; for full panels of experiments to be completed in the time that it took to run an experiment on a single cell previously. Furthermore, more electrophysiological information can be obtained for ion channels of interest from a single cell by using oscillating voltage protocols; an 8-s-long protocol using three additive sine functions can be used to describe the time and voltage dependence of a specific current. This protocol allows for greater data collection in an experimental window and leads to higher replicability to provide more information of current kinetics^196^ but has not yet been used in CMs. In the future, multiple cells of a microtissue may be simultaneously characterized *in situ* using existing high-throughput robotic patch clampers to examine mechanical or structural effects on electrophysiology.^197^ The inability to patch-clamp for long periods of time remains an issue for this technique, as longitudinal experiments on single cells are not possible.

For simplified single-cell voltage transients, or monolayer or tissue estimations of AP speed or intensity, individual live cells and microtissues can be imaged for voltage propagation using voltage-sensitive aminonapthylethenylpyridinium (ANEP) dyes. These dyes, which are largely nonfluorescent in solution, become fluorescently active when incorporated into the cell membrane by their hydrophobic tails. Both dyes are commonly used in CM applications; the general-purpose di-4-ANEPPS is easier to use in new cell types or when developing a protocol in lab, while di-8-ANEPPS can be more difficult to optimize but offers a more stable measurement over time due to its longer hydrophobic tail. These dyes are extremely responsive, reacting to changes in voltage in less than 1 *μ*s. This speed in detection can be capitalized upon by using a line-scanning camera in certain applications; otherwise, the rate of optical capture may limit the interpretation of data. These dyes show relatively small changes in fluorescence per unit voltage change and so are typically normalized to background fluorescence. A full discussion of voltage indicator choice and use can be found elsewhere.^198^

### A. Multielectrode arrays

For monolayer electrophysiological experimentation, especially in high-throughput drug screening, the most popular modality currently lies in MEAs. MEAs are composed of one or more recording and reference electrodes per well, covered by a glass substrate; these electrodes are typically composed of gold or platinum and can be additionally directly covered with a composite such as indium tin oxide for protection and enhanced cell adhesion.^199^ Since their inception, MEAs have been used extensively in proarrhythmia electrophysiological assays through analysis of their field potentials;^29,90,109,116,118,204–206^ detailed discussion of physiological interpretations from MEAs can be found in other reviews.^57^

MEA-derived field potentials can be used directly with little processing to determine simple proarrhythmogenicity; however, their waveform is significantly different from that of a true action potential due to intrinsic capacitances and resistances at the interfaces of the cell and the media. As a result, it is difficult to use a field potential to interpret specific changes to the intensity or duration of each AP phase. Work is underway to interpret field potentials in the context of an MEA-enabled circuit to extrapolate action potential characteristics.^22^ The commercial MEA field is populated by standout platforms including xCELLigence (ACEA Biosciences), CardioExcyte 96 (Nanion Technologies), MED64 (Alpha MED Scientific), and Maestro APEX (Axion Biosystems). The xCELLigence and CardioExcyte 96 platforms are designed for multiplexed measurements of field potential with correlative impedance analysis of contractility; xCELLigence offers electrical field stimulation, while CardioExcyte 96 carries optogenetic stimulation capacity for use with engineered cells. MED64 allows for high signal-to-noise ratio field potential readings with high-current stimulation capability, while the Maestro APEX integrates a HEPA-filtered incubator and robotic liquid handling with optogenetic stimulation for field potential readings. MEAs are currently limited by variability between electrodes, as well as issues of CM subtype purity. They require concerted beating and different isolation, differentiation, and culture protocols between labs which makes MEA results subject to difficulties in reproducibility.^57^ Currently, there is a high false-positive rate of torsadogenic compounds,^203^ suggesting that further analytical development and algorithm training are necessary to produce validated assays.

The solution to current issues in electrophysiology may be found in a marriage between patch-clamping techniques and MEAs, combining the precision and replicability of the former with the ease of use and high-throughput capability of the latter. Arrays of gold nanoposts upon which CMs were seeded were initially developed to measure membrane potentials after electroporation.^204^ These arrays were improved with a switch to hollow iridium oxide electrodes, which allowed for megaohm seals and the use of electrode filling solutions.^205^ This modality was recently extended to allow for electrophysiological voxelization on a microtissue level by using a CMOS array of 32 × 32 pixels, each consisting of 1 *μ*m-spaced nanoneedles.^206^ This device allows for the tissue-level measurement of APs and can construct propagation maps and could prove extremely useful in characterizing co-cultures and fibrillation models. Developments to enhance spatial, temporal, and signal resolution are ongoing; recent improvements can distinguish CM subtypes and certain disease phenotypes.^207^ Although these patch-clamping array modalities hold great promise for CM characterization, they require further development to enhance CM viability for longitudinal experimentation and to characterize the effect of needle topography and nanoelectrode electroporation on the physiological function.^207^

## IX. CALCIUM TRANSIENT MEASUREMENT

The cyclical flux of Ca^2+^ through the cytosol, as discussed above, is vital to CM homeostasis and maintains cell attachments, metabolism, and housekeeping and survival functions.^7,82,153,208–210^ In allowing actomyosin movement through its binding of troponin, Ca^2+^ enables contraction, and the measurement of its transients allows for important insights into its physiological function. The traditional way to measure intracellular calcium flux is by the use of a whole-cell voltage-clamp to specifically measure I_Ca._^197^ Alternatively, Ca^2+^ flux can be measured optically. The development and applications of genetic and chemical indicators of calcium release are excellently reviewed elsewhere.^198,211–213^ Transient amplitude and kinetics, as well as focal Ca^2+^ sparks, or release events from individual RyR clusters, are often assessed.^214^ The general downside of optical measurements of voltage changes and calcium transients, similar to patch-clamp modalities, is that the high-intensity light used to image cells, as well as loading protocols that often require membrane permeabilization, can prove damaging to cells and prevent the longitudinal study.

Two-photon systems have traditionally been limited in resolution and capture rate. However, there have been steady development processing capabilities and spatial depth and resolution^215^ and capture speed to the point of recording Ca^2+^ spark or transient events,^216^ as well as voltage flux or specific biochemical reactions,^217^ could be exploited for enhanced characterization especially in microtissues. Furthermore, the low-energy excitation photons used for two-photon microscopy would minimize photobleaching and damage to the cell.

## X. MEASUREMENT OF CONTRACTILITY

The dynamics of a contracting cardiomyocyte are arguably the most important functional measurements when evaluating a drug or tissue construct. Changes in contractile force production may be due to physiological changes in the myocardial structure, cell morphology, changes to calcium flux, or cytoskeletal organization. Contractility can be measured on a single-cell scale or in isolated or cultured tissues. A single adult cardiomyocyte produces approximately 5 *μ*N of peak isometric force,^136^ while neonatal CMs and PSC-CMs can be matured to produce between 15 and 600 nN each.^100,218,219^ The isometric contractile force generation of adult myocardium *in vivo* ranges from ca. 15 to 60 mN mm^−2^, reaching an average peak of 40–45 mN mm^−2^, which is prone to decrease up to 50% or more in HF or similar phenotypes.^220,221^ Moreover, healthy myocardial peak isometric force development scales positively with stimulation frequency until a very high beat rate (ca. 150–180 bpm in humans) is reached, while pathologic myocardium does not and often decreases in force production even from relatively low beat rates onward.^221,222^ Peak isometric force production is similar between humans and their commonly used experimental surrogates of rats, mice, and rabbits; however, the rates of contraction scale inversely to animal size, at the cost of efficiency.^220^ On the scale of microtissues, engineered constructs recapitulate the *in vivo* cross-sectional force of native tissues better than on an individual cell scale, ostensibly due to enhanced physiological maturation of the composite CMs.

Contraction force can be measured mechanically, optically, or acoustically. The method of the contractility measurement depends on the goals of the experiment and the culture system used. Whenever contractility is being assessed, there should be consideration on how the modality may affect contractility; changes in the cell morphology, limited attachment points, low volumes of media, and non-physiological mechanical loading (in magnitude or directionality) are all potential complications depending on the modality used.^223^ Finally, many previous comparisons between studies have not included considerations between isometric and isotonic measurements, which represent different aspects of CM contractile physiology.^224^ Previous authors have reviewed the available contractility tools and guiding principles of the field;^225^ the focus of this section will be on *in vitro* myocardial applications, current limitations, and opportunities for improvement of the technology.

### A. Measured contractile force production in single cells

Depending on the type of single-cell contractile measurement desired, isometric or isotonic force transducers are available with temporal resolution of at least ca. 250 Hz^223^ and the ability to resolve a force to a sub-micronewton range.^226^ The contractile measurements of single cells can either be live, and contracting spontaneously or under electrical field stimulation, or “skinned cardiomyocyte” preparations, whereupon the cell membrane is permeabilized with detergent and the cell perfused with gradients of Ca^2+^ and ATP.^227^ The latter preparation is not physiologically relevant *per se*; the information offered from a permeabilized cell experiment is more structural than physiological in that it conveys information about cytoskeletal organization and therefore can mechanistically describe the actomyosin complex or morphological changes in the cell. However, the preparation does not incorporate conductance or modulation of excitation-contraction coupling enabled by the intricate calcium-handling infrastructure of the cell.^223^

Isotonically contracting cells exert a traction force on their substrate, which can be estimated by observing point displacements in the region of interest and calculating force production based on the mechanical properties of the substrate in question. Cell-level traction force is most commonly measured by traction force microscopy (TFM) or by analysis of substrate micropost deformation using beam-bending theory. In-depth analysis of both of these methods is available.^228^ Traction force microscopy (TFM) is typically performed on cells adhered to a compliant gel substrate (typically 3–20 kPa) of polyacrylamide or PDMS, coated with an attachment factor. The substrate includes markers, typically fluorescent beads of 0.05–0.5 *μ*m, which have been adhered to the surface of the gel or incorporated within it during polymerization. TFM makes use of a 2-D displacement field calculated between a two-timepoint image stack of the greatest and least contraction magnitude. Contracting cells pull up away from their substrate at edges and push down in their centre,^229^ but on a flat substrate of sufficient thickness, little error is made in only calculating horizontal forces.^230^ Using a material with a Poisson ratio of near 0.5 further decouples vertical from horizontal deflection. Polyacrylamide and PDMS gels typically have Poisson's ratios near ca. 0.48^231^ and 0.5,^232^ respectively, making these approximations quite suitable.

The TFM displacement field is typically constructed by either blockwise image correlations (Particle Image Velocimetry or PIV)^233^ or by the tracking of individual beads between images (Particle Tracking Velocimetry or PVT).^234^ A traction force field is typically derived from the displacement field and solved using established methods.^233,235,236^ Further spatial resolution can be obtained if necessary using image registration at the cost of increased computation.^237^ TFM has been successfully performed in neonatal^238,239^ and PSC-CMs.^24,100,141,240^ For the increased physiological insight, traction reconstruction with point forces (TRPFs), another TFM scheme, improves accuracy and limits model complexity by limiting traction forces to the focal adhesions generated between cells and their substrates. This techniques usually requires live-staining of a focal adhesion-specific protein, such as GFP-vinculin.^241^ TRPF also requires other considerations, namely, the identification of focal adhesion translocation during contraction, the potential for which can vary from cell to cell and from substrate to substrate.^241^ It is however unlikely that fatty acid translocation will significantly confound measurements taken within a contraction.^242^ Advancements are steadily being made to improve accuracy and deconvolute image processing in TRPF.^243^

Beam-bending models can also be used to calculate an individual cell's traction force. Micropost substrates have often been used for measuring contractile force production in single cells,^244–247^ including in CMs.^218,248^ By measuring deflection of each beam, the total force production of a cell can be estimated. Moreover, if each beam is assessed as a single point, each one is independent from the others, and so, coupling effects are not considered. However, the limited attachment points for the cell may affect its morphology and therefore the effect change in mechanical signaling to downstream force production. To this end, micropillars may also be replaced with smaller substrate features; Li *et al.* recently developed a marriage between the “bed of nails” beam-bending technique and TFM, whereupon fluorescent-tipped nanowire displacement was used to compute traction force with sub-nanonewton precision.^249^ The regularity of the substrates facilitates identification of points between images, and the use of the linear elasticity theory also minimizes computation. Although this technique would be useful in its current form for certain applications in PSC-CM physiology, fabrication methods would need to be adapted to the micronewton levels of force produced by more mature CMs. This technique has also been adapted to a high-throughput marriage with TFM, whereupon the deformation of many cell-size patterns each by an individual cell can be used to increase experimental power.^250^

The field of TFM is developing rapidly both in acquisition and interpretation techniques. In addition to the application of improved algorithms for more expedient and accurate analysis, considerable progress is being made in optical capture. As TFM resolution is limited by the spatial sampling frequency and not by the frame rate, the application of stimulated emission depletion (STED) super resolution microscopy to TFM has been successful.^251,252^ The image capture rate of advanced STED systems is sufficient to resolve CM contractions, albeit with extensive post-processing to handle the calculations required at such a spatial resolution. 3D TFM is possible using existing cell-in-gel systems for low-speed TFM^234,253^ or high-speed line-scanning edge detection of CMs,^137^ but optically capturing a Z-stack at sufficient spatial and temporal resolutions during a contraction to allow for TFM is not feasible. With CMs, a 3D modality is less important than other cell types considering the anisotropy of their contraction.

**FIG. 4.**
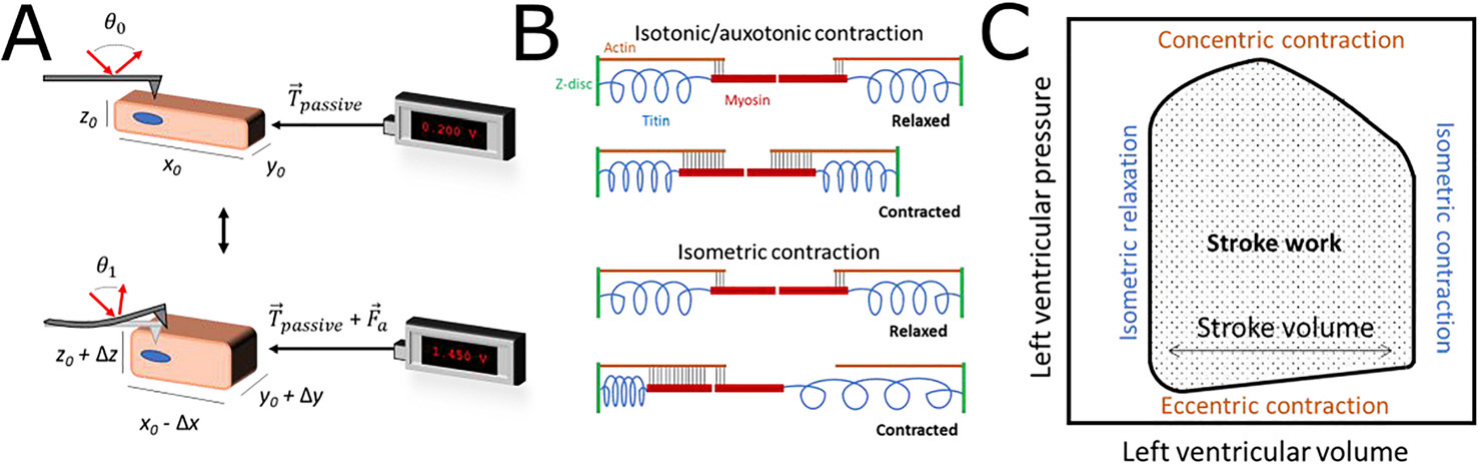
(a) Common principles underlying most measurements of contraction; an axial force can be measured directly using an isometric or strain gauge force transducer. Conversely, during an isotonic contraction, perpendicular cell thickening can be used as a correlative measurement due to the conservation of volume within a contracting cell. The titin structure within the CM offers a baseline of passive resistance ($\vec{T}$_passive_), to which the applied force vector from active contraction ($\vec{F}$_a_) is added. (b) Principles of contraction within a sarcomere of stable A-band dimensions that allow for sarcomeric shortening measurements of isotonic (above) and isometric contraction (below) such as seen in TEM studies.^263^ The scale bar on the left is 1 *μ*m. (c) The heart makes use of both near-isotonic (auxotonic) and isometric contractions with each cycle, as illustrated by a standard pressure-volume loop. The cycle occurs in a counter-clockwise direction, with systole and diastole ending before isometric phases at the top-left and bottom-right corners of the loop, respectively. Passive diastolic filling at near-constant pressure results in eccentric contraction in the myocardium, which corresponds to the degree of preload or end-diastolic pressure.

Most cells are virtually incompressible with a measured Poisson ratio very close to 0.5 for fibroblasts,^254^ which has been habitually extrapolated to CMs in the relevant literature. A consequence of this property is that compression on a certain axis (e.g., longitudinal contraction) will be proportional to expansion in perpendicular axes such that cell volume is maintained. Most modalities to quantify cell contraction without the use of a direct transducer take advantage of this property [Fig. 4(a)]. However, due to their cytoskeletal structure, CMs are most likely orthotropic as opposed to isotropic, and their elastic properties are most likely dynamic over the course of a contraction. The simplest way to measure deflection perpendicular to the axis of contraction of a single cell or microtissue is through the use of atomic force microscopy (AFM) or similar modalities. AFM is routinely used for the measurement of contractility^226,255,256^ but has also been used as a physical stimulus.^257^ The advantages of these modalities are spatial and temporal sensitivity. However, they require repeated calibration, and absolute AFM measurements of elastic modulus tend to differ between studies, suggesting that they should be used primarily for relative comparisons.

Contractile force production can also be estimated by optical measurements of the change in CM or sarcomere dimensions taken from a video of isotonic contraction. Measurements made through these modalities often have the advantage of being non-invasive and can be multiplexed for real-time, physiologically correlated measurements of different metrics, such as live stains and voltage and Ca^2+^ flux.^23,258–260^ Furthermore, these measurements can be made in complex microtissues, assuming that certain mechanical tissue parameters are known.^260^ Contraction amplitude and velocity over time can be plotted and calibrated with reasonable certainty if used in conjunction with force-measuring modalities.^260^ However, there are disadvantages to these modalities. First, they typically require high spatial and temporal resolution together. Second, while several of these tools are compatible with both single cells and tissues, they require regularly shaped objects with clearly defined edges and are not always compatible with complex tissues, immature cells, or opaque bioreactors, Finally, there is often variability between algorithms or measurements made with subjective correction factors, and estimations of contractile force may differ from more direct, albeit invasive, methods.^100^ Certain analysis packages are compatible with high-speed videos taken from mobile phones used in conjunction with microscopes.^240,260^ Edge detection can be improved using fluorescent beads with a single cell,^137^ which can also allow for improved whole-tissue tracking when embedded within a microtissue.^219^A similar modality uses fractional sarcomere shortening as a correlative metric of force production.^21,24,134,261^ Individual sarcomeres are spaced approximately 1.6–2.2 *μ*m apart depending on cell maturity and shorten proportionally to the rest of the cell during a contraction. Sarcomeres can be imaged in brightfield or fluorescent modes, using Lifeact^181^ or similar live stains, or GFP-conjugate expression to better define sarcomeres. Another modality uses diffraction of laser light through the I-bands of sarcomeres which allows for rapid and precise detection of sarcomere shortening by measuring the change in the projected interference pattern by reducing the I-band length over a cell shortening event.^262^ This modality could also be in theory used in isometric contraction due to displacement of the A-band towards one Z-disk [Fig. 4(b)],^263^ although the molecular mechanisms underlying contractile physiology are the subject of continued discussion.^264^

Typical assumptions of whole-cell methods include net directional forces equal to 0 and that the cell of interest is rectangular with perfectly linear and anisotropic contractions. The former assumption is usually justified. However, the latter is reasonable for highly mature cells but not for developing PSC- or neonatal CMs, where developed and force-generating myofibrils are often not perfectly aligned with the major cell axis.

### B. Measurement of contractile force production in monolayer tissues

Changes in tissue impedance during a contraction can be used to monitor beating activity parameters, usually by using an MEA. These assays work by the same principle as other cell thickening modalities: a contracting cell will change its area in the x/y plane and its thickness in the z-axis and will physically interact differently with the substrate. A MEA will measure a change in impedance proportional to the degree of cell movement during contraction.^265,266^ However, as this impedance depends on multiple co-occurring changes that are impossible to separate, changes in impedance allow for the measurement of frequency but only a relative beating intensity. The advantage of this system is the ability for simultaneous high-throughput measurements in different conditions; the commercial xCELLigence MEA system (ACEA Biosciences) for CM culture allows for simultaneous impedance recordings every 12.9 ms in up to 48 wells at once. In general, these MEA systems represent a remarkable improvement in the ability to predict cardiotoxicity in a standardized, high-throughput system,^63^ as they provide a real-time quantitative cell response without the need for expensive biochemical or imaging assays at multiple timepoints. Commercial or homemade MEAs can be further combined with external field potential recordings for electrophysiological monitoring (as previously discussed in Sec. VIII A) and pacing systems to allow for maximal experimental control.^267^ However, the addition of electrical field pacing can complicate multiplexed field potential analysis and generate toxic substances through electrolysis; optogenetic stimulation may enhance these high-throughput multiplexed measurements.^116–118^ Furthermore, contractile analysis using MEAs has been plagued by impaired replicability between wells and the need for manual and subjective data analysis.^57^ Improvements in data analysis are promising increased statistical power and signal recognition.^268,269^

Finally, deflection in the Z-axis has recently been measured through refraction changes detected in cells plated on a diffraction grating to track beat rate and amplitude.^140^ Given the role of nanogrooves in mechanically maturing PSC-CMs, such a modality may prove useful for combined tissue growth and monitoring roles. Furthermore, this technique allows for monitoring of heterogeneous beating patterns across a field of view, which may be of interest for use in enhancing currently existing arrhythmia assays.^53^ Similarly, acoustic microscopy (AM) has been used to measure deflection perpendicular to the axis of contraction, assuming cell incompressibility during contraction. AM modalities such as scanning AM (SAM) use a high-frequency ultrasound transducer with validated subcellular spatial resolution and microsecond-scale temporal resolution,^254,270^ suitable to detect rapid cellular displacements that could in theory be used to measure contraction. Due to its speed of capture, AM can also be used to record an area to estimate spatial heterogeneity in displacement across the cell and simultaneously estimate different viscoelastic properties in real-time.^270–272^ Finally, the setup allows for the optical-free measurement, so that opaque bioreactors or media can be used. Non-scanning AM has been used exploratorily in assessing CM beating; the quartz crystal microbalance technique with dissipation monitoring (QCM-D) has been used to characterize functional chronotropy and mechanical properties of CMs.^273,274^ QCM-D is not sensitive to CM-induced electric field changes, preventing artifacts in the measurement. However, these techniques require an integrated AM transducer in the plate and require a larger cell cluster, limiting their utility in complex systems or single cells. Furthermore, these techniques cannot immediately be used to measure inotropy and lose sensitivity as the cluster is immersed in liquid. These drawbacks could be eliminated by developing SAM for application in CM cultures.

### C. Measurement of contractile force production in engineered cardiac microtissues

The relatively high force produced by engineered microtissues allows for increased resolution and ease of measurement. Isometric or isotonic force production by engineered microtissues can be measured using force transducers. The isotonic force production of engineered microtissues can also be measured through the construct's deflection of a perpendicular anchored cantilever, according to Euler-Bernoulli beam theory. These beams are often constructed of PDMS and incorporated in the bioreactor used to grow and condition the tissue, with their movement sensed by optical or magnetic modalities.^107,108,275^ The beam can also take the form of a cell culture substrate that is anchored at only one end of the axis of contraction; microtissue shortening will curl the cantilever in much the same way.^276^ In the latter study, the cantilever was equipped with a soft strain gauge for enhanced utility in bioreactor design and usage. This is a convenient technique, allowing for a functional measurement that is accessible by optical microscopy and amenable to most culture paradigms.

### D. Passive mechanical measurements

Passive mechanics provide yet another source of physiological information about cells, especially mechanically active CMs. The stiffness of whole isolated cells has been shown to provide important clues as to the disease state of the source heart, ostensibly due to changes in cytoskeletal composition^66^ which would also render the cells elastically anisotropic. Moreover, the balance of evidence suggests that cell stiffness may change during a contraction.^66,277^

Basic AFM-enabled mechanical measurements are being complemented by the development of new modalities capable of more specialized metrics. Novel microelectromechanical systems of various designs could allow for detailed anisotropic measurements in various physiological states of single CMs or microtissues.^278^ The mechanical state of the nucleus (stiffness and mechanical anisotropy) has been shown to also be indicative of cellular phenotype; novel intracellular AFM^279^ and magnetic microtweezer-enabled measurements^280^ could be applied to specifically probe the nucleus or other organelles.

### E. Future directions in mechanical measurements

Constant advancements in tissue culture, microfabrication, and signal analysis promise improvements in existing modalities to quantify contractile force production. In the future, conformationally sensitive Förster resonance energy transfer (FRET) sensors incorporated in titin or other mechanical proteins could potentially be used to quantify shortening kinetics, especially in microtissues.^281^ A 3D characterization of the full traction force field is theoretically possible using existing culture systems;^137^ however, capture time is an issue. In the case of CMs, this modality is likely not especially useful as contraction is primarily unidirectional. However, advanced microtissue characterization may benefit from such capabilities.

Possibly the most significant change to all direct and correlative force measurement modalities will be in incorporating new findings about mechanical loading-specific physiological responses in CMs into culture and measurement techniques. Optical cell shortening measurements are occasionally made using cells in suspension, especially in isolated primary adult CMs. Alternatively, cell shortening measurements in CMs from all sources are often taken from cells cultured on plastic or glass substrates. Care must be taken when comparing these results to other studies as physiological loading and mechanotransduction affect Ca^2+^ handling and force production,^136,137,238,257^ and force sensing occurs due to transduction by titin,^122^ stretch-activated ion channels,^123^ focal adhesions,^120,121^ and intercalated discs.^80,81^ Many recent neonatal and PSC-derived microtissues incorporate passive loading and resistance in their construction. However, many fractional shortening measurements and Ca^2+^ handling assays in adult CMs are done in suspension, which means that results may not reflect *in vivo* realities.^223^ Similarly, there is ongoing debate about the merits and utility of measuring isotonic versus isometric contraction, as these measurements are not directly comparable;^223,224^ a functioning heart will contract isometrically, concentrically, and eccentrically during a beat cycle [Fig. 4(c)]. The modality chosen to measure contractile force production in single cells or tissues must therefore be suited for the phenomenon of interest.

## XI. *IN SILICO* MODELING

Equally important to the building and physicochemical probing of myocardial models is the interpretation that allows for the construction of robust and predictive mathematical models, such that the laboratory testing of every pharmaceutical eventuality is not necessary. These *in silico* models allow for hypothesis generation, economical experimentation, the rigorous and evidence-based development of clinical guidelines, and the advancement of the state of physiology in their own right.

Given the identification of ventricular arrhythmias as one of the most significant contributors to sudden cardiac death (SCD), most *in silico* modeling of pharmaceutical effects on cardiac electrophysiology has focused on the ventricular action potential. The O'Hara-Rudy (ORd) model^282^ was selected by an expert panel as the basis for future development for CiPA-based regulatory frameworks. Detailed discussion of the iterative development of these models is out of the scope of this review; in general, models are gaining traction in successfully recapitulating experimentally observed data and researchers aim to gain further predictive ability in their models in the near future. Current issues being addressed include advanced thermodynamic considerations,^283,284^ hormonal modulation such as beta-adrenergic signaling,^285^ kinetic effects from nonmyocyte populations such as fibroblasts in electrophysiological coupling,^286,287^ and the integration of microstructural models^288^ to allow for whole-myocardial modeling. Most directly important for bioengineers and physiologists, insights originating from *in silico* models can be used to design new experiments, as seen in the novel electrophysiological characterization protocols that provide a far higher degree of AP parameters during an 8-s reading.^289,290^ In general, *in silico* modeling efforts benefit from continued experimentation into the factors underlying ion channel kinetics and drug responses.

## XII. CONCLUSIONS AND FUTURE DIRECTIONS

In the search for the ideal CM model for disease modeling and pharmaceutical testing, preliminary efforts have made it clear that extensive functional characterization of single cells and engineered constructs will be necessary for true understanding of the underlying physiology to improve patient outcomes. Further recapitulation of CM physiology *in vitro* would offer unprecedented resolution, economy of experimentation, and experimental power, but our current understanding of individual CM function is as yet insufficient for full prediction of the cardiac response to disease or pharmaceuticals. It is clear that advancements in cell physiology, culture systems, analysis, and *in silico* predictions will be necessary to provide realistic solutions to the current challenges in therapeutic development. Therefore, convergences between physiology, electrical/computer and mechanical engineering, and physics offer an exciting chance to advance the field.
